# Gene therapy and bone marrow transplantation

**DOI:** 10.1186/1471-2334-14-S2-S19

**Published:** 2014-05-23

**Authors:** Jan Van Lunzen

**Affiliations:** 1 University Medical Center Hamburg-Eppendorf, Hamburg, Germany

## 

Despite the tremendous advances in antiretroviral combination therapy over the last decade, eradication of HIV from the infected organism is still an elusive goal. Lifelong therapy is associated with potential long-term toxicity, adherence problems, and development of drug resistance. Thus, gene therapy approaches targeting viral eradication are still attractive. Here a number of studies have failed to show a clear clinical benefit yet. Current approaches were mainly limited by a low number of transduced cells and genotoxicity. The use of new vector systems and the right choice of target cells and improved transduction protocols may overcome these obstacles. Recent reports on the use of newly developed transgenes either allowing for an enrichment of transduced cells by an in vivo selection advantage or restoration of a functional immune system which is resistant to HIV infection nourished the hope for continuous progress in this field. Indeed the intriguing finding that HIV seems to be eradicated in an individual case study after stem cell transplantation with a mutant coreceptor (CCR5 delta 32 deletion) underlines the proof of the concept.

